# Gastric Microbiota Associated with Gastric Precancerous Lesions in *Helicobacter pylori*-Negative Patients

**DOI:** 10.3390/microorganisms13010081

**Published:** 2025-01-03

**Authors:** Han-Na Kim, Min-Jeong Kim, Jonathan P. Jacobs, Hyo-Joon Yang

**Affiliations:** 1 Department of Clinical Research Design and Evaluation, Samsung Advanced Institute for Health Sciences & Technology, Sungkyunkwan University, Seoul 06355, Republic of Korea; hanna147942@gmail.com; 2 Center for Clinical Epidemiology, Research Institute for Future Medicine, Samsung Medical Center, Seoul 06351, Republic of Korea; 3 Samsung Advanced Institute for Health Sciences & Technology, Sungkyunkwan University, Seoul 06355, Republic of Korea; mjeong12.kim@gmail.com; 4 Research Institute for Future Medicine, Samsung Medical Center, Seoul 06351, Republic of Korea; 5 Vatche and Tamar Manoukian Division of Digestive Diseases, Department of Medicine, David Geffen School of Medicine at UCLA, Los Angeles, CA 90095, USA; jjacobs@mednet.ucla.edu; 6 Goodman-Luskin Microbiome Center, David Geffen School of Medicine at UCLA, Los Angeles, CA 90095, USA; 7 Division of Gastroenterology, Hepatology and Parenteral Nutrition, Veterans Administration Greater Los Angeles Healthcare System, Los Angeles, CA 90073, USA; 8 Division of Gastroenterology, Department of Internal Medicine and Gastrointestinal Cancer Center, Kangbuk Samsung Hospital, Sungkyunkwan University School of Medicine, Seoul 03181, Republic of Korea; 9 Medical Research Institute, Kangbuk Samsung Hospital, Sungkyunkwan University School of Medicine, Seoul 03181, Republic of Korea

**Keywords:** gastric precancerous lesions, gastric microbiota, 16S rRNA, *Helicobacter pylori*

## Abstract

Studies on the gastric microbiota associated with gastric precancerous lesions remain limited. This study aimed to profile the gastric mucosal microbiota in patients with *Helicobacter pylori*-negative precancerous lesions. Gastric mucosal samples were obtained from 67 *H. pylori*-negative patients, including those with chronic gastritis (CG), intestinal metaplasia (IM), and dysplasia. The V3–V4 region of the 16S rRNA gene was sequenced and analyzed. No significant difference was observed in the alpha or beta diversity of the gastric microbiota among the groups. However, a taxonomic analysis revealed a significant enrichment of *Lautropia mirabilis* and the depletion of *Limosilactobacillus reuteri*, *Solobacxterium moorei*, *Haemophilus haemolyticus*, and *Duncaniella dubosii* in the IM and dysplasia groups compared to those in the CG group. *Prevotella jejuni* and the genus *Parvimonas* were enriched in the IM group. A predictive functional analysis revealed enrichment of the ornithine degradation pathway in the IM and dysplasia groups, suggesting its role in persistent gastric mucosal inflammation associated with gastric precancerous lesions. The gastric microbiota associated with *H. pylori*-negative gastric precancerous lesions showed an increased abundance of oral microbes linked to gastric cancer and a reduction in anti-inflammatory bacteria. These alterations might contribute to chronic gastric mucosal inflammation, promoting carcinogenesis in the absence of *H. pylori* infection.

## 1. Introduction

Gastric cancer (GC) remains a notable global health challenge and is predicted to be the fifth leading cause of cancer incidence and mortality worldwide as of 2022 [1]. Although early detection can substantially improve survival, organized population-based screening programs are currently established only in Korea and Japan [2,3]. This underscores the importance of early identification and management of patients with gastric precancerous lesions, including chronic atrophic gastritis (CAG), intestinal metaplasia (IM), and gastric dysplasia [4,5]. Notably, the prevalence of gastric precancerous lesions in the general population can reach up to 30% globally [5]. Despite increasing interest in precancerous lesions, substantial knowledge gaps persist, highlighting the need for further investigation into their pathogenesis and clinical implications.

Recent studies have identified dysbiosis within the gastric microbiota in patients with GC, characterized by reduced diversity and significantly altered composition compared to those in individuals without GC [6,7,8,9,10,11,12]. Specifically, the gastric microbiota in patients with GC exhibited an overrepresentation of oral microbes, such as *Streptococcus* and *Fusobacterium*, as well as lactic acid bacteria, including *Lactobacillus* [6,7,8,9,13,14,15]. Although some studies included patients with gastric precancerous lesions as part of the control groups [9,14,15,16], investigations specifically focusing on the microbiota associated with gastric precancerous lesions remain limited [17,18,19]. Investigating the gastric microbiota associated with precancerous lesions is important for understanding the pathogenesis and early microbial changes during gastric carcinogenesis. Particularly, examining the gastric microbiota of patients who are *Helicobacter pylori*-negative is crucial. Gastric precancerous lesions in patients who are *H. pylori*-negative are generally indicative of a past infection, as chronic *H. pylori* infection initiates the gastric carcinogenesis process. However, it is often depleted or lost in GC [7]. Moreover, as *H. pylori* is an infectious agent, eradication therapy is recommended upon its detection [20]. Previous studies had often excluded *H. pylori*-positive cases from analysis due to the profound impact of *H. pylori* on the gastric microbiota [6,18,21]. By profiling the gastric mucosal microbiota of *H. pylori*-negative patients with chronic gastritis (CG), IM, and dysplasia, the current study aimed to uncover the microbial alterations associated with gastric precancerous lesions and explore their potential contributions to carcinogenesis.

## 2. Materials and Methods

### 2.1. Study Participants and Sample Collection

**Figure 1:**
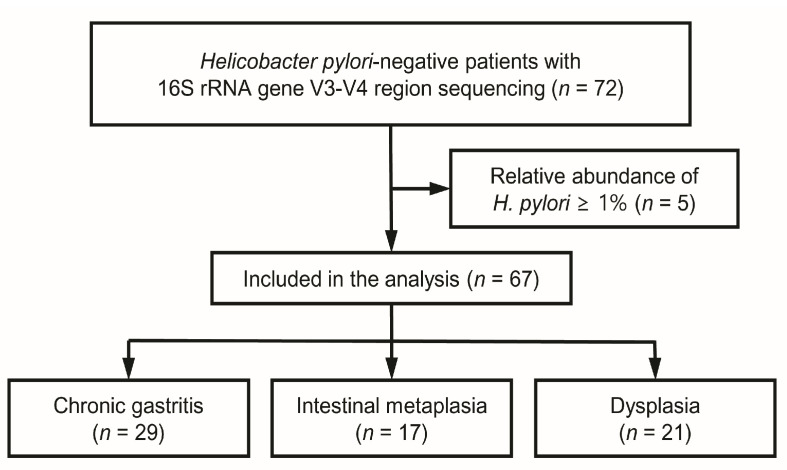
A flowchart of the study.

This study included patients with gastric precancerous lesions who were *H. pylori*-negative (Figure 1). Patients with CAG and IM without gastric dysplasia were classified into the IM group, whereas those with gastric dysplasia in a background CAG and IM were classified into the dysplasia group. The CG group consisted of *H. pylori*-negative individuals with CG and without any precancerous lesion, serving as the control group. The study was part of a broader investigation on the gastric microbiota associated with *H. pylori*-negative GC [6,22]. Patients were recruited from Kangbuk Samsung Hospital, Seoul, Republic of Korea, between April 2020 and April 2021. The exclusion criteria were any recent use of antibiotics, probiotics, proton-pump inhibitors, H_2_ receptor antagonists, mucoprotective agents, or antacids over the past month; a history of *H. pylori* eradication within the past year; or prior gastrectomy.

Endoscopic procedures were performed using a disinfected endoscope, and tissue biopsies for microbiome analysis were obtained from the greater curvature of the mid antrum using sterile forceps [23]. The biopsy tissues were immediately stored at −20 °C, transferred to a −80 °C freezer within 6 h, and stored there until analysis. CAG was endoscopically assessed as mucosal thinning with a pale surface and visible submucosal vessels, whereas IM was histologically confirmed through biopsies of the lesser curvatures of the antrum and body [24]. *H. pylori*-negative status was confirmed through both a negative rapid urease test and modified Giemsa staining, as well as by the relative abundance of *H. pylori* of <1% in microbiota profiling [25].

### 2.2. Ethics

The Institutional Review Board of Kangbuk Samsung Hospital (KBSMC 2020-03-027) approved all study procedures. The consent form for participation was distributed to all participants and signed.

### 2.3. 16S rRNA Gene Sequencing

Genomic DNA was extracted from gastric mucosal biopsy samples using a DNeasy PowerSoil Kit (Qiagen, Hilden, Germany) according to the manufacturer’s protocol. Amplicon libraries targeting the V3–V4 hypervariable region of the bacterial 16S rRNA gene were prepared by polymerase chain reaction (PCR) using universal forward and reverse primers (337F–805R). Bidirectional sequencing (2 × 300 bp) of the libraries was performed using an Illumina MiSeq platform (Illumina Inc., San Diego, CA, USA). Negative controls were included in the DNA extraction and amplification processes. No detectable amplicon was observed after 30 PCR cycles, confirming the absence of contamination. The demultiplexed sequencing data were subsequently denoised using the DADA2 algorithm to identify amplicon sequence variants (ASVs) within the Quantitative Insights Into Microbial Ecology 2 (QIIME2) 2021.4 platform. Taxonomic classification was performed by referencing the National Center for Biotechnology Information Nucleotide and Taxonomy database (NCBI-RefSeq, accessed 9 June 2021).

### 2.4. Microbial Diversity and Taxonomic Analysis

For diversity metrics, sequence counts were rarefied to 11,160 reads per sample to standardize the sequencing depth across samples. The alpha diversity was assessed using several metrics, including the total number of observed features, Pielou’s evenness, Shannon index, and Faith’s phylogenetic diversity, calculated using QIIME2. The diversity indices were exported and compared across the CG, IM, and dysplasia groups using an analysis of covariance (ANCOVA), adjusted for age and sex, in R (version 4.0.0; R Foundation for Statistical Computing, Vienna, Austria).

Beta diversity was evaluated by calculating the distance matrices for Bray–Curtis dissimilarity, Jaccard distance, unweighted UniFrac distance, and weighted UniFrac distance using QIIME2. The resulting matrices were exported and analyzed to compare the bacterial community composition across the CG, IM, and dysplasia groups, adjusted for age and sex. A permutational multivariate analysis of variance (PERMANOVA) with 10,000 permutations was conducted using the adonis function in R [26]. A principal coordinate analysis (PCoA) was performed using the pcoa function in R, and the data were visualized using 95% data ellipses to represent group dispersion.

For taxonomic comparison, taxa with a prevalence of less than 15% were filtered out [27]. Differentially abundant taxa between the IM and CG groups, or the dysplasia and CG groups, from phylum to species levels, were identified using the DESeq2 function in R, adjusted for age and sex. The DESeq2 models count data with a negative binomial distribution to allow for normalization, shrinkage of dispersion estimates, and hypothesis testing to detect significant differences between the conditions [28]. Statistical significance for multiple testing was corrected using the Benjamini–Hochberg method, with an adjusted false discovery rate (FDR) *q*-value < 0.1 considered significant.

### 2.5. Functional Profiling Using PICRUSt2

Microbial community functions were predicted from the amplicon sequences using the Phylogenetic Investigation of Communities by Reconstruction of Unobserved States 2 (PICRUSt2) software (v.2.5.3) [29]. Phylogenetic placement in PICRUSt2 involves three key steps, namely the hidden Markov models (HMMER) to place ASVs, an evolutionary placement algorithm (EPA-NG) to determine the best placement of these ASVs within a reference phylogeny, and genesis applications for phylogenetic placement analysis (GAPPA) to generate a tree of the most likely ASV placements [29]. The process generated a phylogenetic tree containing both reference genomes and environmentally sampled organisms, which was subsequently used to predict the individual gene family copy numbers for each ASV. Gene family predictions were annotated using enzyme classification (EC) numbers and aggregated into pathway-level abundance predictions based on the MetaCyc database [30]. The MetaCyc pathway abundance tables generated by the PICRUSt2 built-in mapping tool were used for downstream statistical analyses.

Differential abundance analysis (DAA) of the MetaCyc pathways was conducted using the DESeq2 algorithm in the R statistical environment (version 2024.09.0+375). The model included adjustments for age and sex as covariates to account for potential confounding effects. Log_2_ fold changes in the pathway abundances between groups were calculated, and the *p*-values were adjusted for multiple comparisons using the Benjamini–Hochberg (BH) method. Significantly enriched pathways were identified based on an adjusted *q*-value threshold of FDR < 0.1.

## 3. Results

A total of 72 patients were enrolled in this study (Figure 1). After excluding five patients who exhibited a relative *H. pylori* abundance greater than 1% in the microbial analysis, 67 *H. pylori*-negative patients were included in the final analysis (29 patients with CG, 17 with IM, and 21 with dysplasia). The patient demographics are summarized in Table 1. The median ages (interquartile range) were 44 (33–53.5), 59 (53.5–70), and 65 (55.5–72) years in patients with CG, IM, and dysplasia, respectively (*p* < 0.001). However, no significant differences were observed for sex or BMI across the three groups. To profile the gastric mucosal microbiota, 16S rRNA gene sequencing of the V3–V4 region was performed using gastric biopsies. In total, 2571 ASVs were identified from an average of 45,476 reads across 67 samples after data processing.

### 3.1. Gastric Mucosal Microbial Diversity Did Not Significantly Differ in H. pylori-Negative Precancerous Lesions than That in Controls

**Figure 2:**
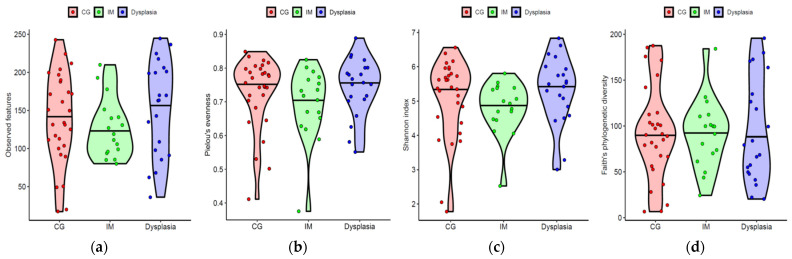
Alpha diversity in the gastric microbiota did not significantly differ among the chronic gastritis (CG), intestinal metaplasia (IM), and dysplasia groups. (**a**) Observed amplicon sequence variants, (**b**) Pielou’s evenness, (**c**) Shannon index, and (**d**) Faith’s phylogenetic diversity. Horizontal lines indicate medians. No significant difference was found among the groups for any alpha diversity index after adjusting for age and sex.

To determine the changes in gastric mucosal microbial diversity associated with precancerous lesions, we compared the alpha diversity across the CG, IM, and dysplasia groups (Figure 2). The alpha diversity indices, including the observed microbial features, microbial evenness, Shannon diversity, and phylogenetic diversity, were not significantly different among the CG, IM, and dysplasia groups (all *p* > 0.05, Mann–Whitney U test), and the results did not change after adjusting for age and sex (all *p* > 0.05, ANCOVA).

**Figure 3:**
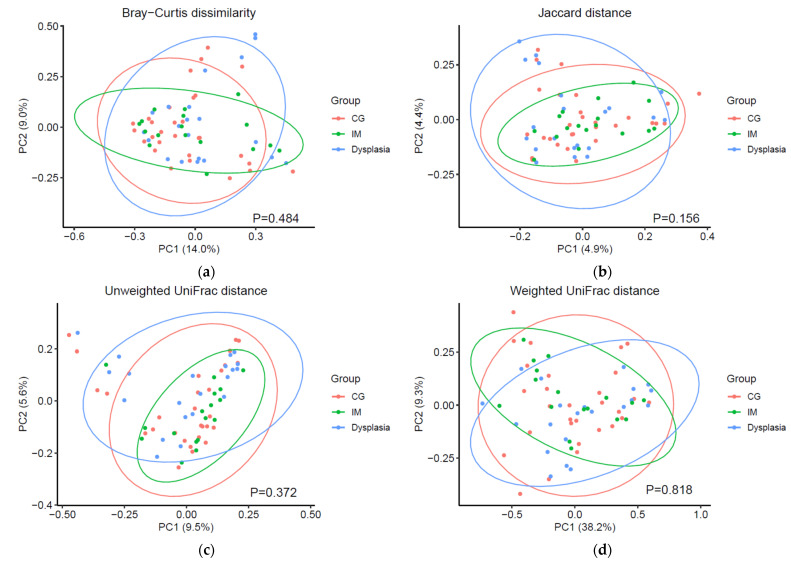
Overall gastric microbiota composition did not significantly differ between the chronic gastritis (CG), intestinal metaplasia (IM), and dysplasia groups in beta diversity analysis. (**a**) Bray–Curtis dissimilarity, (**b**) Jaccard distance, (**c**) unweighted UniFrac distance, and (**d**) weighted UniFrac distance. Visualization was performed using principal coordinate analysis. Effect size and significance were assessed using permutational multivariate analysis of variance with 10,000 permutations, adjusting for age and sex. Ellipses represent 95% of data points for each group.

The differences in microbial composition across the CG, IM, and dysplasia groups were computed and visualized in PCoA plots (Figure 3). In the unadjusted PERMANOVA analyses, no significant differences were observed among the CG, IM, and dysplasia groups across multiple metrics, including Bray–Curtis dissimilarity (*p* = 0.316), Jaccard distance (*p* = 0.590), unweighted UniFrac distance (*p* = 0.799), and weighted UniFrac distance (*p* = 0.550). After adjusting for age and sex, the results remained similar (all *p* > 0.05). The lack of significant differences in diversity suggested that alterations in specific taxa, rather than global diversity shifts, play a dominant role in the progression of precancerous lesions.

### 3.2. Gastric Mucosal Microbiota of Patients with Gastric Precancerous Lesions Were Characterized by Taxonomic Alterations

**Figure 4:**
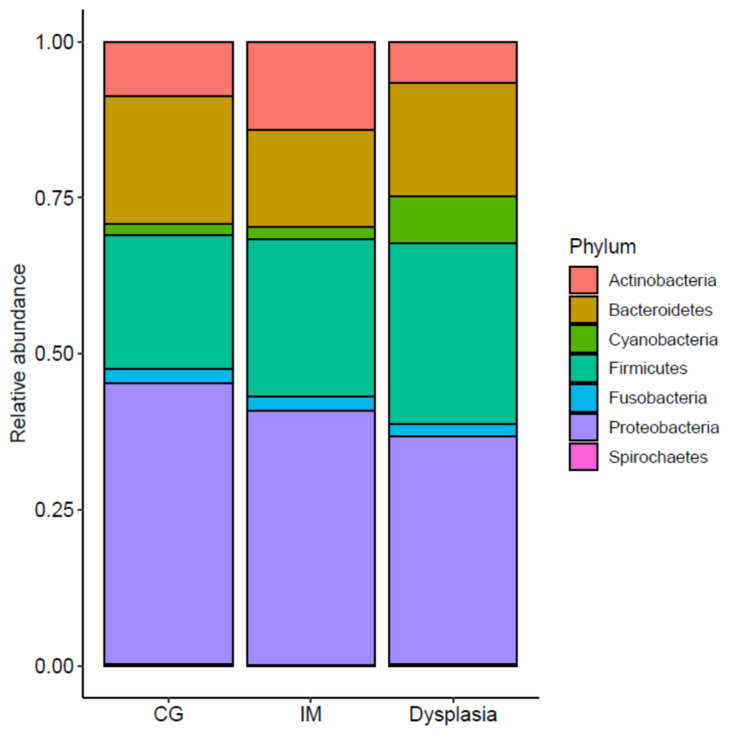
Gastric mucosal microbiota did not differ at the phylum level among the chronic gastritis (CG), intestinal metaplasia (IM), and dysplasia groups. Although a trend of decreasing relative abundance of Proteobacteria and increasing relative abundance of Firmicutes was observed from the CG to the IM and dysplasia groups, no statistically significant difference was observed among the groups by DESeq2 analysis after adjusting for age and sex.

**Figure 5:**
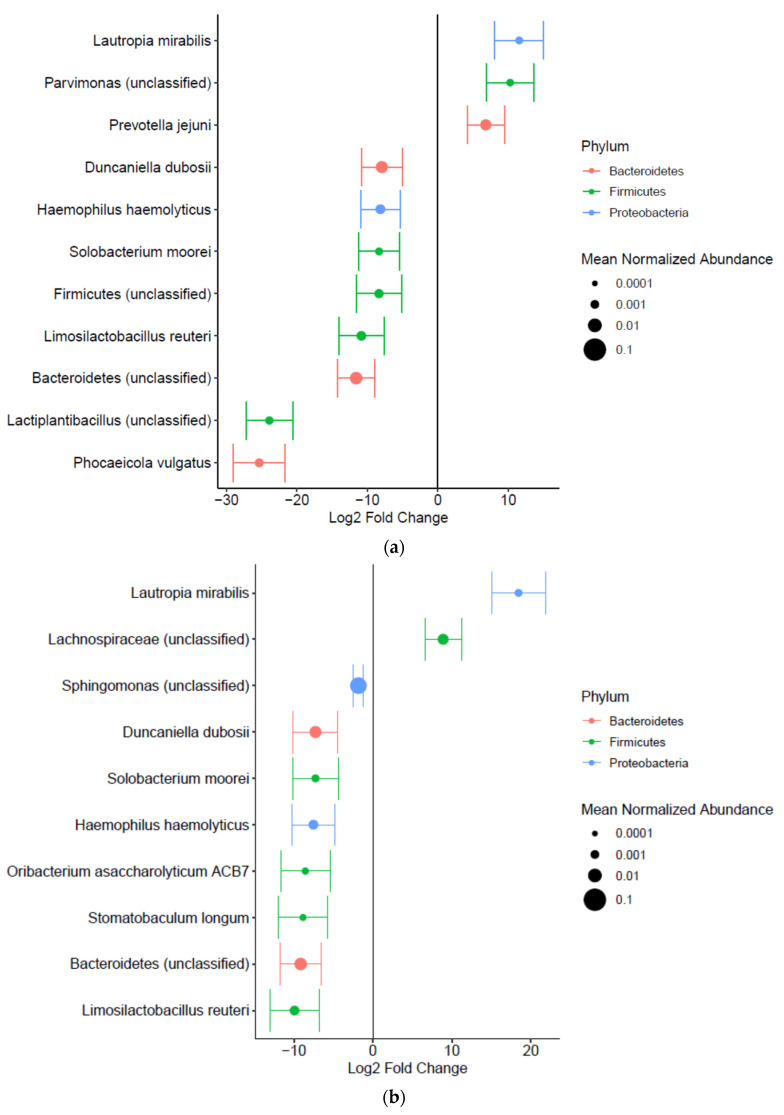
Differentially abundant taxa were identified in patients with gastric precancerous lesions compared to those without. Differentially abundant taxa are shown for (**a**) the comparison between intestinal metaplasia (IM) and chronic gastritis (CG) groups and (**b**) the comparison between dysplasia and CG groups, identified at the species level using DESeq2 analysis. The effect size represents log_2_ fold changes between the groups with 95% confidence intervals. Dot size reflects normalized relative abundance, and colors indicate corresponding phyla. Statistical significance was determined as a false discovery rate *q*-value < 0.1, with adjustment for age and sex.

The taxonomic alterations associated with precancerous lesions were evaluated using the DESeq2 method with adjustments for age and sex. Proteobacteria, Firmicutes, and Bacteroidetes were the most abundant phyla across all groups (Figure 4). Although a trend of decreasing relative abundance of Proteobacteria and increasing relative abundance of Firmicutes was observed from the CG to the IM and dysplasia groups, the changes were not statistically significant, and no differentially abundant taxon were found between the groups at this level. However, at more specific taxonomic levels, 27 taxa (seven enriched and 20 depleted) in the IM group and 19 taxa (5 enriched and 14 depleted) in the dysplasia group showed differential abundance across taxonomic ranks from class to species when compared to the CG group, after adjusting for age and sex (Table S1). Notably, *Lautropia mirabilis* and its higher-order genus *Lautrophia* were enriched in both the IM and dysplastic groups (FDR *q* < 0.1) (Figure 5). In addition, *Prevotella jejuni* and an unclassified species of genus *Parvimonas*, as well as the genus *Parvimonas*, were enriched in the IM group, whereas unclassified species of the family Lachnospiraceae were enriched in the dysplasia group (FDR *q* < 0.1). Conversely, *Limosilactobacillus reuteri*, *Solobacterium moorei*, *Haemophilus haemolyticus*, *Duncaniella dubosii*, and an unclassified species from the phylum Bacteroidetes were depleted in both the IM and dysplasia groups (FDR *q* < 0.1).

### 3.3. PICRUSt2 Analysis Identified the Functional Profile Associated with Gastric Dysplasia

Functional profiling of the gastric microbiota was performed using PICRUSt2 to predict the MetaCyc pathway abundance associated with gastric precancerous lesions. Differential abundance analysis revealed several pathways that were significantly enriched or depleted in the IM and dysplasia groups than those in the CG group after adjustment for age and sex (Table S2). In the IM group, the superpathway of ornithine degradation (ORNDEG-PWY, log_2_FC = −26.39, FDR *q* = 2.93 × 10⁻^7^), which is a key pathway in polyamine metabolism, a process implicated in mucosal cell proliferation and inflammation, was depleted. Further, we observed depletion of the chorismate biosynthesis II (archaea) pathway (PWY-6165, log_2_FC = −20.22, FDR *q* = 3.13 × 10⁻^4^), a precursor pathway for aromatic amino acid synthesis, and a superpathway for mycolyl–arabinogalactan–peptidoglycan biosynthesis (PWY-6404, log_2_FC = −21.72 FDR *q* = 7.29 × 10⁻^5^), reflecting differing alterations in microbial cell wall synthesis in the IM group than that in the CG group. Additionally, the superpathway of sulfolactate degradation (PWY-6641) showed depletion in the IM group (log_2_FC = −11.99, FDR *q* = 8.33 × 10⁻^3^). In the dysplasia group, the superpathway of ornithine degradation (ORNDEG-PWY, log_2_FC = −25.55, FDR *q* = 4.96 × 10⁻^7^) was significantly depleted, indicating its persistent reduction in advanced stages of gastric lesion progression.

## 4. Discussion

In this study, we investigated the gastric microbiota profiles of *H. pylori*-negative patients with gastric precancerous lesions. Our analysis found no significant difference in the alpha or beta diversity of the gastric microbiota among the CG, IM, and dysplasia groups. However, a taxonomic analysis identified specific taxa associated with *H. pylori*-negative gastric precancerous lesions. Notably, the gastric microbiota in both the IM and dysplasia groups were characterized by the enrichment of *Lautropia mirabilis* and the depletion of *Limosilactobacillus reuteri*, *Solobacterium moorei*, *Haemophilus haemolyticus*, and *Duncaniella dubosii*. Additionally, the enrichment of *Prevotella jejuni* and the genus *Parvimonas* was associated with gastric IM.

The results of our study suggested that the gastric microbiota associated with precancerous lesions can be characterized more effectively through taxonomic comparisons rather than by diversity analyses. We observed a significant increase in the relative abundance of *Lautropia mirabilis* in the gastric microbiota of patients with IM and dysplasia who were *H. pylori*-negative compared to those with CG. *Lautropia*, one of the oral microbes [31] that had previously been reported to be enriched in patients with GC or precancerous lesions [32]. It is also associated with periodontal disease [33] and cystic fibrosis [34], as well as with the oral microbiota of children with human immunodeficiency virus infection [35]. Further, we identified an increased abundance of other oral microbes, namely *Prevotella jejuni* and *Parvimonas*, in the gastric microbiota of patients with IM. *Prevotella* has been reported to be overrepresented in patients with *H. pylori*-negative CAG [18], gastric dysplasia [36], and GC [36,37], whereas *Parvimonas* has similarly been associated with GC [16]. Additionally, Lachnospiraceae, which was found to be enriched in gastric dysplasia in our study, is a known producer of short-chain fatty acids [38] and has been implicated in both gastric and colorectal cancers [39]. These findings suggested that oral microbes, such as *Lautropia mirabilis*, *Prevotella jejuni*, and *Parvimonas*, survive within the altered microenvironment of gastric premalignant lesions, where reduced acidity allows their persistence. Their presence may contribute to the development of gastric precancerous lesions by inducing chronic inflammation in the gastric mucosa. Additionally, Lachnospiraceae may play a role in modulating this environment by supplying short-chain fatty acids, which may further influence gastric carcinogenesis.

Another significant taxonomic alteration associated with gastric precancerous lesions was the reduced abundance of bacteria with known anti-inflammatory effects. Our analysis showed a significant depletion of *Limosilactobacillus reuteri*, *Haemophilus haemolyticus*, and *Duncaniella dubosii* in both the IM and dysplasia groups. *Limosilactobacillus reuteri* is a well-known probiotic with beneficial effects, including the production of anti-inflammatory mediators [40]. *Haemophilus* exhibits nitrate-reducing capabilities and has been shown to mitigate chronic inflammation [41]. Our previous study identified a reduced abundance of *Haemophilus* in *H. pylori*-negative patients with GC [6]. *Duncaniella dubosii* plays a protective role in experimental models of colitis induced by dextran sulfate sodium [42,43]. These findings implied that a reduction in anti-inflammatory microbes may contribute to the progression of gastric precancerous lesions in the absence of an ongoing *H. pylori* infection.

An intriguing finding was the decreased abundance of *Solobacterium moorei* in patients with gastric precancerous lesions, as this bacterium is commonly associated with halitosis [44] and opportunistic infections in the oral cavity [45,46]. This observation might parallel the paradox of *H. pylori*, which gradually depletes over the course of gastric carcinogenesis, despite initiating the process [8]. Further studies would be required to validate these findings and clarify the role of *Solobacterium moorei* in the development of gastric precancerous lesions.

Functional profiling of the gastric microbiota revealed a significant depletion of key metabolic pathways in both the IM and dysplasia groups compared to that in the CG group. Ornithine degradation was significantly reduced in the IM and dysplasia groups. Ornithine metabolism plays a crucial role in enhancing gut barrier function by upregulating tight junction protein expression and mitigating inflammatory responses in immune cells [47]. A reduction in gut microbiome-driven ornithine metabolism has been linked to an increased susceptibility to *Clostridioides difficile* infection [48]. These observations suggested that gastric mucosal microbiota other than *H. pylori* contribute to persistent gastric mucosal inflammation during gastric carcinogenesis.

The association between gastric microbial diversity and precancerous lesions remains debatable. Although several studies have reported no significant decrease in microbial diversity in patients with gastric precancerous lesions compared to that in controls [10,16,17,37,49,50,51,52], others have suggested a reduction [9,12,14,18,21,36], particularly in cases of gastric dysplasia [14,18,21,36]. Analyses excluding *H. pylori*-positive cases have shown decreased diversity in patients with gastric dysplasia [18]. Even among meta-analyses, the findings remain inconsistent. One meta-analysis observed a decline in the alpha diversity in patients with CAG and IM compared to those with superficial gastritis [11], whereas another, encompassing the largest cohort to date, found no significant difference in alpha diversity between patients with gastritis and IM after removing *H. pylori* sequences [10]. In our study, we specifically examined the differences in alpha diversity across the CG, IM, and dysplasia groups in *H. pylori*-negative cases and observed no significant difference. Our findings are consistent with those of the aforementioned large meta-analysis, suggesting that changes in alpha diversity in the gastric microbiota of patients with gastric precancerous lesions are minimal once the impact of *H. pylori* is excluded.

Furthermore, our data indicated no significant difference in beta diversity across the CG, IM, and dysplastic groups. Unlike that of alpha diversity, previous studies that included patients with gastric precancerous lesions and cancers did not report the results of direct significance testing of beta diversity differences between patients with precancerous lesions and controls [12,14,16,21,36,37,49,50,52]. Although some studies have identified significant differences in microbial composition between these groups [18,51], others, including a study using shotgun metagenomics, have not observed such differences [9,17]. A systematic review found significant compositional differences between patients with IM and those with gastritis in a large pooled cohort after excluding *H. pylori* sequences [10]. However, this difference was the smallest among the comparisons involving GC stages, such as between gastritis and GC or IM and GC. Collectively, the findings suggested that the differences in gastric microbial composition among patients with CG, IM, and dysplasia are relatively minor and may only become detectable in sufficiently large sample sizes.

A key strength of our study is its focus on the gastric microbiota specifically in patients with gastric precancerous lesions, an area that has received less attention compared to GC despite its clinical significance. Another notable strength is the inclusion of *H. pylori*-negative patients, which enabled us to explore the role of the gastric microbiota in precancerous lesions independent of *H. pylori* infection. While eradication therapy is often performed in *H. pylori*-positive patients to reduce the risk of GC, limited information is available on GC development in those who have undergone prior *H. pylori* treatment. Our findings highlight the potential of metagenomics and metabolomics as tools for detecting gastric precancerous lesions in *H. pylori*-negative patients.

This study also has several limitations. First, the relatively modest sample size may have limited our ability to detect subtle differences in gastric microbial diversity between patients with and without gastric precancerous lesions. Second, healthy controls might have been a more appropriate control group than individuals with CG included in our study. However, CG is a common finding during upper endoscopy in asymptomatic individuals without an increased risk of GC. Most studies on gastric microbiota have used patients with chronic or superficial gastritis as the control group [6,8,9,11,14,18,19,21], although some have included healthy individuals [15,16]. Third, the cross-sectional design precluded causal inference, as the temporal relationship between microbiota changes and lesion development could not be established. Fourth, our study was conducted exclusively on the Korean population, which could limit the generalizability of our findings to other ethnic or geographic groups. Additionally, the study lacked data on smoking history and past medical history, other than gastric surgery, which were therefore not included in the adjusted analysis. Finally, the functional profiles of the gastric microbiota were inferred from 16S rRNA sequencing data. Although predictive functional analyses, such as those performed using PICRUSt2, provided valuable insights, they rely on reference databases and cannot completely capture the functional activity of the microbial community. Therefore, these results should be interpreted with caution and regarded as hypotheses awaiting further validation.

Future studies employing more comprehensive approaches, such as shotgun metagenomics or metabolomics, would be required to confirm the functional predictions and elucidate the metabolic interactions between the gastric microbiota and the host. Future directions could also include studies utilizing non-invasive samples, such as saliva or feces, to identify the metagenomic or metabolomic biomarkers associated with gastric precancerous lesions or cancer.

In conclusion, the current study profiled and compared the gastric microbiota of *H. pylori*-negative patients with and without gastric precancerous lesions. The microbiota associated with *H. pylori*-negative gastric precancerous lesions was found to be characterized by an increased abundance of oral microbes linked to GC and a reduction in anti-inflammatory bacteria. Predictive functional analysis revealed a depletion of the ornithine degradation pathway, a process associated with the mitigation of gut inflammatory processes, in patients with precancerous lesions. Microbial alterations might contribute to chronic gastric mucosal inflammation and promote gastric carcinogenesis in the absence of *H. pylori* infection.

## Figures and Tables

**Table 1:** Demographics of patients included in the study.

	Chronic Gastritis (*n* = 29)	Intestinal Metaplasia (*n* = 17)	Dysplasia (*n* = 21)	*p*-Value
Age, years, median (interquartile range)	44 (33–53.5)	59 (53.5–70)	65 (55.5–72)	<0.001
Sex, *n* (%)				0.183
Female	18 (62.1)	7 (41.2)	8 (38.1)	
Male	11 (37.9)	10 (58.8)	13 (61.9)	
Body mass index, *n* (%)				0.799
<25 kg/m^2^	23 (79.3)	12 (70.6)	16 (76.2)	
≥25 kg/m^2^	6 (20.7)	5 (29.4)	5 (23.8)	

Kruskal–Wallis and chi-squared tests were used to compare the three groups.

## Data Availability

The original sequencing data generated and analyzed for this study will be made available on request from the corresponding author due to ethical reasons.

## Supplementary Materials

The following supporting information can be downloaded at https://www.mdpi.com/article/10.3390/microorganisms13010081/s1: Table S1: Differentially abundant taxa in gastric precancerous lesions identified using DESeq2 analysis; Table S2: Differentially abundant MetaCyc pathways in gastric precancerous lesions identified using PICRUSt2 and DESeq2 analyses.

## References

[1] Bray F., Laversanne M., Sung H., Ferlay J., Siegel R.L., Soerjomataram I., Jemal A. (2024). Global cancer statistics 2022: GLOBOCAN estimates of incidence and mortality worldwide for 36 cancers in 185 countries. CA Cancer J. Clin..

[2] Mabe K., Inoue K., Kamada T., Kato K., Kato M., Haruma K. (2022). Endoscopic screening for gastric cancer in Japan: Current status and future perspectives. Dig. Endosc..

[3] Jun J.K., Choi K.S., Lee H.Y., Suh M., Park B., Song S.H., Jung K.W., Lee C.W., Choi I.J., Park E.C. (2017). Effectiveness of the Korean National Cancer Screening Program in Reducing Gastric Cancer Mortality. Gastroenterology.

[4] Dinis-Ribeiro M., Shah S., El-Serag H., Banks M., Uedo N., Tajiri H., Coelho L.G., Libanio D., Lahner E., Rollan A. (2024). The road to a world-unified approach to the management of patients with gastric intestinal metaplasia: A review of current guidelines. Gut.

[5] Marques-Silva L., Areia M., Elvas L., Dinis-Ribeiro M. (2014). Prevalence of gastric precancerous conditions: A systematic review and meta-analysis. Eur. J. Gastroenterol. Hepatol..

[6] Kim H.N., Kim M.J., Jacobs J.P., Yang H.J. (2022). Altered gastric microbiota and inflammatory cytokine responses in patients with *Helicobacter pylori*-negative gastric cancer. Nutrients.

[7] Zeng R., Gou H., Lau H.C.H., Yu J. (2024). Stomach microbiota in gastric cancer development and clinical implications. Gut.

[8] Ferreira R.M., Pereira-Marques J., Pinto-Ribeiro I., Costa J.L., Carneiro F., Machado J.C., Figueiredo C. (2018). Gastric microbial community profiling reveals a dysbiotic cancer-associated microbiota. Gut.

[9] Coker O.O., Dai Z., Nie Y., Zhao G., Cao L., Nakatsu G., Wu W.K., Wong S.H., Chen Z., Sung J.J.Y. (2018). Mucosal microbiome dysbiosis in gastric carcinogenesis. Gut.

[10] Li Y., Hu Y., Zhan X., Song Y., Xu M., Wang S., Huang X., Xu Z.Z. (2023). Meta-analysis reveals Helicobacter pylori mutual exclusivity and reproducible gastric microbiome alterations during gastric carcinoma progression. Gut Microbes.

[11] Liu D., Zhang R., Chen S., Sun B., Zhang K. (2022). Analysis of gastric microbiome reveals three distinctive microbial communities associated with the occurrence of gastric cancer. BMC Microbiol..

[12] Liu C., Ng S.K., Ding Y., Lin Y., Liu W., Wong S.H., Sung J.J., Yu J. (2022). Meta-analysis of mucosal microbiota reveals universal microbial signatures and dysbiosis in gastric carcinogenesis. Oncogene.

[13] Castano-Rodriguez N., Goh K.L., Fock K.M., Mitchell H.M., Kaakoush N.O. (2017). Dysbiosis of the microbiome in gastric carcinogenesis. Sci. Rep..

[14] Park J.Y., Seo H., Kang C.S., Shin T.S., Kim J.W., Park J.M., Kim J.G., Kim Y.K. (2022). Dysbiotic change in gastric microbiome and its functional implication in gastric carcinogenesis. Sci. Rep..

[15] Pimentel-Nunes P., Barros A., Pita I., Miranda I., Conceicao G., Borges-Canha M., Leite-Moreira A.F., Libanio D., Dinis-Ribeiro M. (2021). Gastric microbiome profile throughout gastric carcinogenesis: Beyond helicobacter. Scand. J. Gastroenterol..

[16] Gantuya B., El Serag H.B., Matsumoto T., Ajami N.J., Uchida T., Oyuntsetseg K., Bolor D., Yamaoka Y. (2020). Gastric mucosal microbiota in a Mongolian population with gastric cancer and precursor conditions. Aliment. Pharmacol. Ther..

[17] Wu F., Yang L., Hao Y., Zhou B., Hu J., Yang Y., Bedi S., Sanichar N.G., Cheng C., Perez-Perez G. (2022). Oral and gastric microbiome in relation to gastric intestinal metaplasia. Int. J. Cancer. J. Int. Du. Cancer.

[18] Liu D., Chen S., Gou Y., Yu W., Zhou H., Zhang R., Wang J., Ye F., Liu Y., Sun B. (2021). Gastrointestinal Microbiota Changes in Patients With Gastric Precancerous Lesions. Front. Cell Infect. Microbiol..

[19] Park C.H., Lee A.R., Lee Y.R., Eun C.S., Lee S.K., Han D.S. (2019). Evaluation of gastric microbiome and metagenomic function in patients with intestinal metaplasia using 16S rRNA gene sequencing. Helicobacter.

[20] Malfertheiner P., Megraud F., Rokkas T., Gisbert J.P., Liou J.M., Schulz C., Gasbarrini A., Hunt R.H., Leja M., O’Morain C. (2022). Management of *Helicobacter pylori* infection: The Maastricht VI/Florence consensus report. Gut.

[21] Sun Q.H., Zhang J., Shi Y.Y., Zhang J., Fu W.W., Ding S.G. (2022). Microbiome changes in the gastric mucosa and gastric juice in different histological stages of Helicobacter pylori-negative gastric cancers. World J. Gastroenterol. WJG.

[22] Kim M.J., Kim H.N., Jacobs J.P., Yang H.J. (2024). Combined DNA Methylation and Gastric Microbiome Marker Predicts Helicobacter pylori-Negative Gastric Cancer. Gut Liver.

[23] Yang H.J., Seo S.I., Lee J., Huh C.W., Kim J.S., Park J.C., Kim H., Shin H., Shin C.M., Park C.H. (2023). Sample Collection Methods in Upper Gastrointestinal Research. J. Korean Med. Sci..

[24] Yang H.J., Kang D., Chang Y., Ahn J., Ryu S., Cho J., Guallar E., Sohn C.I. (2020). Diabetes mellitus is associated with an increased risk of gastric cancer: A cohort study. Gastric Cancer.

[25] Kim J., Kim N., Jo H.J., Park J.H., Nam R.H., Seok Y.J., Kim Y.R., Kim J.S., Kim J.M., Kim J.M. (2015). An Appropriate Cutoff Value for Determining the Colonization of Helicobacter pylori by the Pyrosequencing Method: Comparison with Conventional Methods. Helicobacter.

[26] McArdle B.H., Anderson M.J. (2001). Fitting multivariate models to community data: A comment on distance-based redundancy analysis. Ecology.

[27] Cao Q., Sun X., Rajesh K., Chalasani N., Gelow K., Katz B., Shah V.H., Sanyal A.J., Smirnova E. (2020). Effects of rare microbiome taxa filtering on statistical analysis. Front. Microbiol..

[28] Love M.I., Huber W., Anders S. (2014). Moderated estimation of fold change and dispersion for RNA-seq data with DESeq2. Genome Biol..

[29] Douglas G.M., Maffei V.J., Zaneveld J.R., Yurgel S.N., Brown J.R., Taylor C.M., Huttenhower C., Langille M.G.I. (2020). PICRUSt2 for prediction of metagenome functions. Nat. Biotechnol..

[30] Caspi R., Billington R., Fulcher C.A., Keseler I.M., Kothari A., Krummenacker M., Latendresse M., Midford P.E., Ong Q., Ong W.K. (2018). The MetaCyc database of metabolic pathways and enzymes. Nucleic Acids Res..

[31] Chen T., Yu W.H., Izard J., Baranova O.V., Lakshmanan A., Dewhirst F.E. (2010). The Human Oral Microbiome Database: A web accessible resource for investigating oral microbe taxonomic and genomic information. Database.

[32] Peng X., Yao S., Huang J., Zhao Y., Chen H., Chen L., Yu Z. (2023). Alterations in bacterial community dynamics from noncancerous to Gastric cancer. Front. Microbiol..

[33] Papapanou P.N., Park H., Cheng B., Kokaras A., Paster B., Burkett S., Watson C.W., Annavajhala M.K., Uhlemann A.C., Noble J.M. (2020). Subgingival microbiome and clinical periodontal status in an elderly cohort: The WHICAP ancillary study of oral health. J. Periodontol..

[34] Ben Dekhil S.M., Peel M.M., Lennox V.A., Stackebrandt E., Sly L.I. (1997). Isolation of Lautropia mirabilis from sputa of a cystic fibrosis patient. J. Clin. Microbiol..

[35] Rossmann S.N., Wilson P.H., Hicks J., Carter B., Cron S.G., Simon C., Flaitz C.M., Demmler G.J., Shearer W.T., Kline M.W. (1998). Isolation of Lautropia mirabilis from oral cavities of human immunodeficiency virus-infected children. J. Clin. Microbiol..

[36] Wang Z., Gao X., Zeng R., Wu Q., Sun H., Wu W., Zhang X., Sun G., Yan B., Wu L. (2020). Changes of the gastric mucosal microbiome associated with histological stages of gastric carcinogenesis. Front. Microbiol..

[37] Zhang X., Li C., Cao W., Zhang Z. (2021). Alterations of Gastric Microbiota in Gastric Cancer and Precancerous Stages. Front. Cell Infect. Microbiol..

[38] Vacca M., Celano G., Calabrese F.M., Portincasa P., Gobbetti M., De Angelis M. (2020). The Controversial Role of Human Gut Lachnospiraceae. Microorganisms.

[39] Su Q., Jin C., Bo Z., Yang Y., Wang J., Wang J., Zhou J., Chen Y., Zeng H., Chen G. (2023). Association between gut microbiota and gastrointestinal cancer: A two-sample bi-directional Mendelian randomization study. Front. Microbiol..

[40] Abuqwider J., Altamimi M., Mauriello G. (2022). Limosilactobacillus reuteri in Health and Disease. Microorganisms.

[41] Raubenheimer K., Bondonno C., Blekkenhorst L., Wagner K.H., Peake J.M., Neubauer O. (2019). Effects of dietary nitrate on inflammation and immune function, and implications for cardiovascular health. Nutr. Rev..

[42] Feng P., Li Q., Liu L., Wang S., Wu Z., Tao Y., Huang P., Wang P. (2022). Crocetin Prolongs Recovery Period of DSS-Induced Colitis via Altering Intestinal Microbiome and Increasing Intestinal Permeability. Int. J. Mol. Sci..

[43] Chang C.S., Liao Y.C., Huang C.T., Lin C.M., Cheung C.H.Y., Ruan J.W., Yu W.H., Tsai Y.T., Lin I.J., Huang C.H. (2021). Identification of a gut microbiota member that ameliorates DSS-induced colitis in intestinal barrier enhanced Dusp6-deficient mice. Cell Rep..

[44] Barrak I., Stajer A., Gajdacs M., Urban E. (2020). Small, but smelly: The importance of Solobacterium moorei in halitosis and other human infections. Heliyon.

[45] Yu S., Wang X., Li Z., Jin D., Yu M., Li J., Li Y., Liu X., Zhang Q., Liu Y. (2024). Solobacterium moorei promotes the progression of adenomatous polyps by causing inflammation and disrupting the intestinal barrier. J. Transl. Med..

[46] Alauzet C., Aujoulat F., Lozniewski A., Ben Brahim S., Domenjod C., Enault C., Lavigne J.P., Marchandin H. (2021). A New Look at the Genus Solobacterium: A Retrospective Analysis of Twenty-Seven Cases of Infection Involving S. moorei and a Review of Sequence Databases and the Literature. Microorganisms.

[47] Rooks M.G., Garrett W.S. (2016). Gut microbiota, metabolites and host immunity. Nat. Rev. Immunol..

[48] Pruss K.M., Enam F., Battaglioli E., DeFeo M., Diaz O.R., Higginbottom S.K., Fischer C.R., Hryckowian A.J., Van Treuren W., Dodd D. (2022). Oxidative ornithine metabolism supports non-inflammatory C. difficile colonization. Nat. Metab..

[49] Li T.H., Qin Y., Sham P.C., Lau K.S., Chu K.M., Leung W.K. (2017). Alterations in gastric microbiota after *H. pylori* eradication and in different histological stages of gastric carcinogenesis. Sci. Rep..

[50] He C., Peng C., Shu X., Wang H., Zhu Z., Ouyang Y., Yang X., Xie C., Hu Y., Li N. (2022). Convergent dysbiosis of gastric mucosa and fluid microbiome during stomach carcinogenesis. Gastric Cancer.

[51] Zhang Y., Shen J., Shi X., Du Y., Niu Y., Jin G., Wang Z., Lyu J. (2021). Gut microbiome analysis as a predictive marker for the gastric cancer patients. Appl. Microbiol. Biotechnol..

[52] Huang K., Gao X., Wu L., Yan B., Wang Z., Zhang X., Peng L., Yu J., Sun G., Yang Y. (2021). Salivary Microbiota for Gastric Cancer Prediction: An Exploratory Study. Front. Cell Infect. Microbiol..

