# Supplementary material for: Gastric Microbiota Associated with Gastric Precancerous Lesions in Helicobacter pylori-Negative Patients
Source: Microorganisms. 2025 Jan 3;13(1):81. doi: 10.3390/microorganisms13010081 (PMC11767925; doi:10.3390/microorganisms13010081)
Supplement: Supplementary file 1 [file microorganisms-13-00081-s001.zip › microorganisms-3377106-supplementary.pdf]

Supplementary Material

Gastric Microbiota Associated with Gastric Precancerous Lesions in *Helicobacter pylori*-Negative Patients

Table S1. Differentially abundant taxa in gastric precancerous lesions identified using DESeq2 analysis

Table S2. Differentially abundant MetaCyc pathways in gastric precancerous lesions identified using PICRUSt2 and DESeq2 analyses

Table S1. Differentially abundant taxa in gastric precancerous lesions identified using DESeq2 analysis

Taxonomic assignment	Group	log_2FC	SE	*p*	FDR *q*
p__Proteobacteria;					
c__Betaproteobacteria;					
o__Burkholderiales;					
f__Burkholderiaceae;					
g__Lautropia;	IM	11.38	3.52	0.001	0.017
s__mirabilis	IM	11.55	3.48	0.001	0.014
p__Firmicutes;					
c__Tissierellia;					
o__Tissierellales;					
f__Peptoniphilaceae;					
g__Parvimonas;	IM	10.78	3.32	0.001	0.017
s__(unclassified)	IM	10.26	3.34	0.002	0.028
p__Bacteroidetes;					
c__Bacteroidia;					
o__Bacteroidales;					
f__Prevotellaceae;					
g__Prevotella;					
s__jejuni	IM	6.81	2.64	0.010	0.072
p__Firmicutes;					
c__Bacilli;	IM	1.74	0.56	0.002	0.019
o__Lactobacillales	IM	1.78	0.59	0.002	0.041
p__Bacteroidetes;					
c__Bacteroidia;					
o__Bacteroidales;					
f__(unclassified)	IM	−7.20	2.79	0.010	0.099
p__Firmicutes;					
c__Clostridia;					
o__Eubacteriales;					
f__Clostridiales	IM	−7.83	2.52	0.002	0.031
p__Bacteroidetes;					
c__Bacteroidia;					
o__Bacteroidales;					
f__Muribaculaceae;					
g__Duncaniella;					
s__dubosii	IM	−7.95	2.89	0.006	0.054
p__Proteobacteria;					
c__Gammaproteobacteria;					
o__Pasteurellales;					
f__Pasteurellaceae;					
g__Haemophilus;					
s__haemolyticus	IM	−8.15	2.81	0.004	0.043
p__Firmicutes;					

c__Erysipelotrichia;					
o__Erysipelotrichales;					
f__Erysipelotrichaceae;					
g__Solobacterium;	IM	−8.42	2.91	0.004	0.043
s__moorei	IM	−8.34	2.94	0.005	0.046
p__Firmicutes;					
c__(unclassified);					
o__(unclassified);	IM	−8.43	3.01	0.005	0.058
f__(classified);	IM	−7.77	3.02	0.010	0.099
g__(unclassified);					
s__(unclassified)	IM	−8.36	3.22	0.009	0.072
p__Firmicutes;					
c__Clostridia;					
o__Eubacteriales;					
f__Lachnospiraceae;					
g__Lachnoanaerobaculum	IM	−8.61	3.14	0.006	0.058
p__Firmicutes;					
c__Clostridia;					
o__Eubacteriales;					
f__Clostridiaceae	IM	−9.06	2.81	0.001	0.031
p__Firmicutes;					
c__Bacilli;					
o__Lactobacillales;					
f__Lactobacillaceae;					
g__Limosilactobacillus;	IM	−10.59	2.66	0.000	0.002
s__reuteri	IM	−10.85	3.21	0.001	0.014
p__Bacteroidetes;					
c__(unclassified);	IM	−10.96	2.60	0.000	0.001
o__(unclassified);	IM	−11.59	2.59	0.000	0.000
f__(unclassified);	IM	−11.76	2.59	0.000	0.000
g__(unclassifieid);					
s__(unclassified)	IM	−11.60	2.61	0.000	0.000
p__Firmicutes;					
c__Bacilli;					
o__Lactobacillales;					
f__Lactobacillaceae;					
g__Lactiplantibacillus;	IM	−23.64	3.30	0.000	0.000
s__(unclassified)	IM	−23.92	3.31	0.000	0.000
p__Bacteroidetes;					
c__Bacteroidia;					
o__Bacteroidales;					
f__Bacteroidales;					
g__Phocaeicola;					
s__vulgatus	IM	−25.36	3.68	0.000	0.000
p__Proteobacteria;					

c__Betaproteobacteria;					
o__Burkholderiales;					
f__Burkholderiaceae;					
g__Lautropia;	Dysplasia	18.57	3.45	0.000	0.000
s__mirabilis	Dysplasia	18.44	3.41	0.000	0.000
p__Firmicutes;					
c__Clostridia;					
o__Eubacteriales;					
f__Lachnospiraceae;					
g__(unclassified);	Dysplasia	8.06	2.23	0.000	0.006
s__(unclassified)	Dysplasia	8.90	2.30	0.000	0.004
p__Proteobacteria;					
c__Gammaproteobacteria	Dysplasia	1.75	0.67	0.009	0.086
p__Proteobacteria;					
c__Alphaproteobacteria;					
o__Sphingomonadales;					
f__Sphingomonadaceae;					
g__Sphingomonas;					
s__(unclassified)	Dysplasia	−1.84	0.65	0.005	0.064
p__Bacteroidetes;					
c__Bacteroidia;					
o__Bacteroidales;					
f__Muribaculaceae;					
g__Duncaniella;					
s__dubosii	Dysplasia	−7.28	2.84	0.010	0.093
p__Firmicutes;					
c__Erysipelotrichia;					
o__Erysipelotrichales;					
f__Erysipelotrichaceae;					
g__Solobacterium;					
s__moorei	Dysplasia	−7.28	2.88	0.012	0.093
p__Proteobacteria;					
c__Gammaproteobacteria;					
o__Pasteurellales;					
f__Pasteurellaceae;					
g__Haemophilus;					
s__haemolyticus	Dysplasia	−7.53	2.76	0.006	0.064
p__Firmicutes;					
c__Clostridia;					
o__Eubacteriales;					
f__Clostridiales dis	Dysplasia	−8.26	2.48	0.001	0.021
p__Firmicutes;					
c__Clostridia;					
o__Eubacteriales;					
f__Lachnospiraceae;					
g__Oribacterium;					

s__asaccharolyticum	Dysplasia	−8.56	3.14	0.006	0.064
p__Firmicutes;					
c__Clostridia;					
o__Eubacteriales;					
f__Lachnospiraceae;					
g__Stomatobaculum;	Dysplasia	−8.70	3.11	0.005	0.073
s__longum	Dysplasia	−8.86	3.16	0.005	0.064
p__Bacteroidetes;					
c__(unclassified);	Dysplasia	−8.29	2.55	0.001	0.024
o__(unclassified);	Dysplasia	−8.35	2.54	0.001	0.034
f__(unclassified);	Dysplasia	−9.20	2.55	0.000	0.015
g__(unclassifieid);					
s__(unclassified)	Dysplasia	−9.15	2.56	0.000	0.010
p__Firmicutes;					
c__Bacilli;					
o__Lactobacillales;					
f__Lactobacillaceae;					
g__Limosilactobacillus;	Dysplasia	−9.47	2.61	0.000	0.006
s__reuteri	Dysplasia	−9.93	3.15	0.002	0.033

The taxa detected to be significantly different in either the IM or dysplasia group than that in the CG group across the six taxonomic levels are included in this table. Taxonomic assignments were performed using the National Center for Biotechnology Information Nucleotide and Taxonomy database (NCBI-RefSeq, accessed on June 9, 2021). $\log_2$ fold changes and false discovery rate (FDR) *q*-values were calculated using DESeq2 with adjustments for age and sex as covariates. The $\log_2$ fold changes were calculated using the CG group as the reference, with positive values indicating enrichment and negative values indicating depletion in the IM and dysplasia groups. CG, chronic gastritis; IM, dysplasia; FC, fold change; SE, standard error; and FDR, false discovery rate.

Table S2. Differentially abundant MetaCyc pathways in gastric precancerous lesions identified using PICRUSt2 and DESeq2 analyses

Pathway	Group	log_2FC	SE	*p*	FDR *q*
superpathway of phenylethylamine degradation (PWY-6071)	IM	−10.02	2.94	<0.001	0.050
3-phenylpropanoate and 3-(3-hydroxyphenyl)propanoate degradation (PWY0-1277)	IM	−11.74	3.71	0.002	0.098
superpathway of sulfolactate degradation (PWY-6641)	IM	−11.99	3.06	<0.001	0.008
chorismate biosynthesis II (archaea) (PWY-6165)	IM	−20.22	4.29	<0.001	<0.001
superpathway of mycolyl-arabinogalactan-peptidoglycan complex biosynthesis (PWY-6404)	IM	−21.72	4.28	<0.001	<0.001
superpathway of ornithine degradation (ORNDEG-PWY)	IM	−26.39	4.29	<0.001	<0.001
chorismate biosynthesis II (archaea) (PWY-6165)	Dysplasia	13.56	4.20	0.001	0.078
L-histidine degradation I (HISDEG-PWY)	Dysplasia	−0.69	0.21	<0.001	0.078
superpathway of sulfolactate degradation (PWY-6641)	Dysplasia	−10.18	3.00	<0.001	0.078
superpathway of phenylethylamine degradation (PWY-6071)	Dysplasia	−10.33	2.89	<0.001	0.067
3-phenylpropanoate and 3-(3-hydroxyphenyl)propanoate degradation (PWY0-1277)	Dysplasia	−11.93	3.64	<0.001	0.078
superpathway of ornithine degradation (ORNDEG-PWY)	Dysplasia	−25.56	4.21	<0.001	<0.001

Log_2 fold changes and false discovery rate *q*-values were calculated using DESeq2 with adjustments for age and sex as covariates. The log_2 fold changes were calculated using the CG group as the reference, with positive values indicating enrichment and negative values indicating depletion in the IM and dysplasia groups. CG, chronic gastritis; IM, dysplasia; FC, fold change; SE, standard error; and FDR, false discovery rate.
